# TRAIL and Taurolidine induce apoptosis and decrease proliferation in human fibrosarcoma

**DOI:** 10.1186/1756-9966-27-82

**Published:** 2008-12-12

**Authors:** Adrien Daigeler, Christina Brenzel, Daniel Bulut, Anne Geisler, Christoph Hilgert, Marcus Lehnhardt, Hans U Steinau, Annegret Flier, Lars Steinstraesser, Ludger Klein-Hitpass, Ulrich Mittelkötter, Waldemar Uhl, Ansgar M Chromik

**Affiliations:** 1 Department of Plastic Surgery, Burn Center, Hand Center, Sarcoma Reference Center, BG-University Hospital Bergmannsheil, Bürkle-de-la-Camp-Platz 1, 44789 Bochum, Germany; 2 Department of General and Visceral Surgery, St. Josef Hospital, Ruhr-University, Gudrunstraße 56, 44791 Bochum, Germany; 3 Department of Medicine II, St. Josef Hospital, Ruhr-University, Gudrunstraße 56, 44791 Bochum, Germany; 4 Institute for Cell Biology (Tumor Research), University of Duisburg-Essen, Virchowstraße 173 45122 Essen, Germany

## Abstract

**Background:**

Disseminated soft tissue sarcoma still represents a therapeutic dilemma because effective cytostatics are missing. Therefore we tested TRAIL and Tarolidine (TRD), two substances with apoptogenic properties on human fibrosarcoma (HT1080).

**Methods:**

Viability, apoptosis and necrosis were visualized by TUNEL-Assay and quantitated by FACS analysis (Propidiumiodide/AnnexinV staining). Gene expression was analysed by RNA-Microarray and the results validated for selected genes by rtPCR. Protein level changes were documented by Western Blot analysis. NFKB activity was analysed by ELISA and proliferation assays (BrdU) were performed.

**Results and discussion:**

The single substances TRAIL and TRD induced apoptotic cell death and decreased proliferation in HT1080 cells significantly. Gene expression of several genes related to apoptotic pathways (TRAIL: *ARHGDIA*, *NFKBIA*, *TNFAIP3*; TRD: *HSPA1A/B*, *NFKBIA*, *GADD45A*, *SGK*, *JUN*, *MAP3K14*) was changed. The combination of TRD and TRAIL significantly increased apoptotic cell death compared to the single substances and lead to expression changes in a variety of genes (*HSPA1A/B*, *NFKBIA*, *PPP1R15A*, *GADD45A*, *AXL*, *SGK*, *DUSP1*, *JUN*, *IRF1*, *MYC*, *BAG5*, *BIRC3*). NFKB activity assay revealed an antipodal regulation of the several subunits of NFKB by TRD and TRD+TRAIL compared to TRAIL alone.

**Conclusion:**

TRD and TRAIL are effective to induce apoptosis and decrease proliferation in human fibrosarcoma. A variety of genes seems to be involved, pointing to the NFKB pathway as key regulator in TRD/TRAIL-mediated apoptosis.

## Background

Fibrosarcoma is a rare entity within the heterogeneous group of soft tissue sarcomas. It accounts for approximately 2.6% of soft tissue sarcomas which themselves have an incidence of about 2–4/100000 [1]. Surgical resection is the key factor in primary treatment and radiation can improve local control, but once the disease has spread, the remaining treatment options are very limited. Response rates to established chemotherapeutic agents like doxorubicin and ifosfamide (with up to 30% at best) are still disappointing [2]. Therefore, new agents are being sought to broaden the therapeutic armament.

TRAIL (tumor necrosis factor receptor apoptosis inducing ligand) has previously been associated with apoptosis in a variety of malignant cells [3] and in HT1080 as well [4]. Whereas FasL (Fas Ligand) and TNF caused significant side effects by unselective apoptogenic effects on normal cells [5], TRAIL proved to be much less toxic and at least equally effective.

Many substances, including established chemotherapeutics like 5-Fluorouracil, cisplatin, doxorubicin, etoposide and others, like vitamime E succinate and alpha-Tocopheryl succinate have been shown to sensitize tumor cells to TRAIL-induced apoptosis [6-9]. Recent studies revealed apoptotic effects of another substance, Taurolidine, that was originally used as an antiinfective in peritonitis. Taurolidine exerted apoptotic activity on a variety of malignant cells in vitro and in vivo [10-12]. First reports of successful treatments of glioblastoma and advanced gastric cancer without systemic side effects in humans are promising [13,14]. Taurolidine has previously been shown to enhance Fas-Ligand mediated cell death [15] and a xenograft study using recombinant TNF in the treatment of mouse fibrosarcoma revealed that Taurolidine reduced the toxicity of TNF without decreasing the anti-tumor efficacy of TNF [16]. The detailed mechanism of action is still unclear, but inhibition of Bcl-2 and an increased efflux of cytochrome-c, an activation of the caspases, and an increased PARP (poly (ADP-ribose) polymerase) cleavage seem to be involved [10,17,18]. By comparison, other authors found Fas-ligand dependent mechanisms or an inhibition of tumor angiogenesis to be responsible for the inhibition of tumor growth [15,19].

In contrast to established chemotherapeutics, the absence of toxicity makes Taurolidine candidate for co-treatment with TRAIL. Inspired by previous studies that showed synergistic effects of TRAIL in combination with Taurolidine inducing apoptotic cell death in human colon and esophageal carcinoma cells [20,21], we examined the effects of these two substances on human fibrosarcoma.

## Methods

### Cell line

Human fibrosarcoma cells, HT1080, were purchased from ATCC (Cell line CCI 121, Wesel, Germany) and maintained with modified Eagle's medium (MEM) and NEAA (non-essential amino acids) + 10% FBS supplemented with 1% penicillin (100 U/ml) and streptomycin (100 μg/ml), 1% Sodium Pyruvate and 1% L-Glutamine. Cells were cultured in a humidified atmosphere with 5% CO_2 _at 37°C in 25 cm^2 ^flasks.

### Reagents

Taurolidine (TRD) (Taurolin^® ^2%, Boehringer Ingelheim, Germany) containing 5% Povidon was used as supplied by the manufacturer. A 5% Povidon solution (K16 Povidon, generously provided by Geistlich Pharma AG, Wolhusen, Switzerland) in equal volume served as control for the TRD group. Recombinant human TRAIL/Apo2L (Bender MedSystems, Vienna, Austria) was dissolved in distilled water according to the manufacturer's instructions. Distilled water in equal volume served as control in the TRAIL experiments.

### Dose-finding study

Cells were incubated with TRD (50, 100, 250, 500 μmol/l) or recombinant human TRAIL (10, 50, 100, 500 ng/ml) and the respective controls (Povidon/H_2_O) for 2, 6, 12, 24 h to identify effective single concentrations and the time dependency of the effects. All experiments were repeated with 3 consecutive passages.

The lowest effective single concentration TRAIL 50 ng/ml that induced apoptosis but no significant necrosis and TRD 250 μmol/l, that showed the highest apoptotic rates and was most effective reducing viable cells were then used as single substances and in combination to identify a possibly synergistic effect. As time points 2, 6, 12, and 24 h were chosen. All experiments were repeated with 3 consecutive passages. Cells for gene expression were harvested after 2 h.

### Flow cytometry analysis

At the indicated incubation time, floating cells were collected together with the supernatant and adherent cells which were harvested by trypsinization. Cells were sedimented by centrifugation, resuspended in 195 μl binding buffer (Bender MedSystems, Vienna, Austria) and incubated with 5 μl Annexin V-FITC (BD Biosciences, Heidelberg, Germany) and 10 μl Propidiumiodide (PI) (Bender MedSystems, Vienna, Austria) following the manufacturer's manual. Cells were analyzed immediately using a FACS flow cytometer (FACS Calibur BD Biosciences, Heidelberg, Germany). For each measurement, 20.000 cells were counted. Dot plots and histograms were analyzed by CellQuest Pro software (BD Biosciences, Heidelberg, Germany). Annexin V positive cells were considered apoptotic; Annexin V and PI positive cells were identified as necrotic. Annexin V and PI negative cells were termed viable.

### Cell morphology

Morphology of adherent cells and cells suspended in culture medium was studied and documented using a phase contrast microscope, Zeiss Axiovert 25 (Karl Zeiss, Jena, Germany).

### TUNEL-assay

Apoptosis was evaluated by terminal deoxynucleotidyl transferase-mediated dUTP-nick end-labeling (TUNEL) using the In Situ Cell Death Detection Kit, Fluorescein (Roche Applied Science, Mannheim, Germany) according to the manufacturer's instructions and analyzed by fluorescence microscopy (Leica DM4000B, Leica Microsystems, Nussloch, Germany).

### Annexin-PI staining for fluorescence microscopy

Cells were incubated and prepared as for the TUNEL assay but stained with 5 μl Annexin V-FITC and 10 μl PI. Photos were taken immediately after staining using fluorescence microscopy (Leica DM4000B, Leica Microsystems, Nussloch, Germany).

### Proliferation-assay

To determine and quantitate the effects of the different substances on cell proliferation, a colorimetric cell proliferation BrdU (5-bromo-2'-deoxyuridine)-ELISA (Roche Applied Science, Mannheim, Germany) was used according to the manufacturer's instructions. Based on the incorporation of the thymidine analogue BrdU during DNA-synthesis, the amount of newly synthesized DNA and thus of proliferation cells is detected using a microplate absorbance reader Sunrise™ (Tecan trading AG, Switzerland) after applying anti-BrdU conjugated with peroxidase (POD) and enhancing a specific substrate reaction. For this experiment, cells were incubated with the different substances for 8 h.

### Statistical analysis

**Figure 1:**
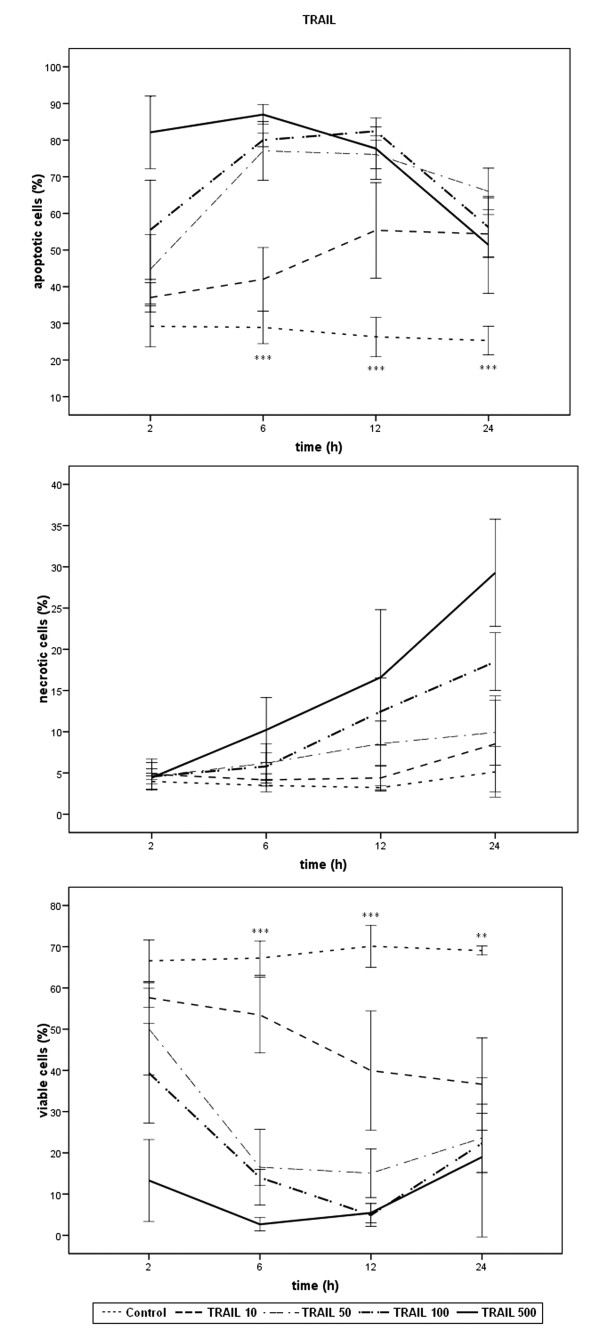
Effects of TRAIL on viability, apoptosis and necrosis in HT1080 cells measured by FACS-analysis: Cells were incubated with TRAIL in the concentrations indicated and with H_2_O (control) for 24 h. The percentages of viable, apoptotic and necrotic cells were determined by FACS-analysis for Annexin V-FITC and Propidiumiodide. Values are means ± SD of 3 independent experiments with consecutive passage (*** p ≤ 0.001, ** p ≤ 0.005; one way ANOVA). The indicators of significance refer to the difference between the 50 ng/ml and the control series. The scales of the y-axis were adjusted to the different values for clarity and therefore vary.

**Figure 2:**
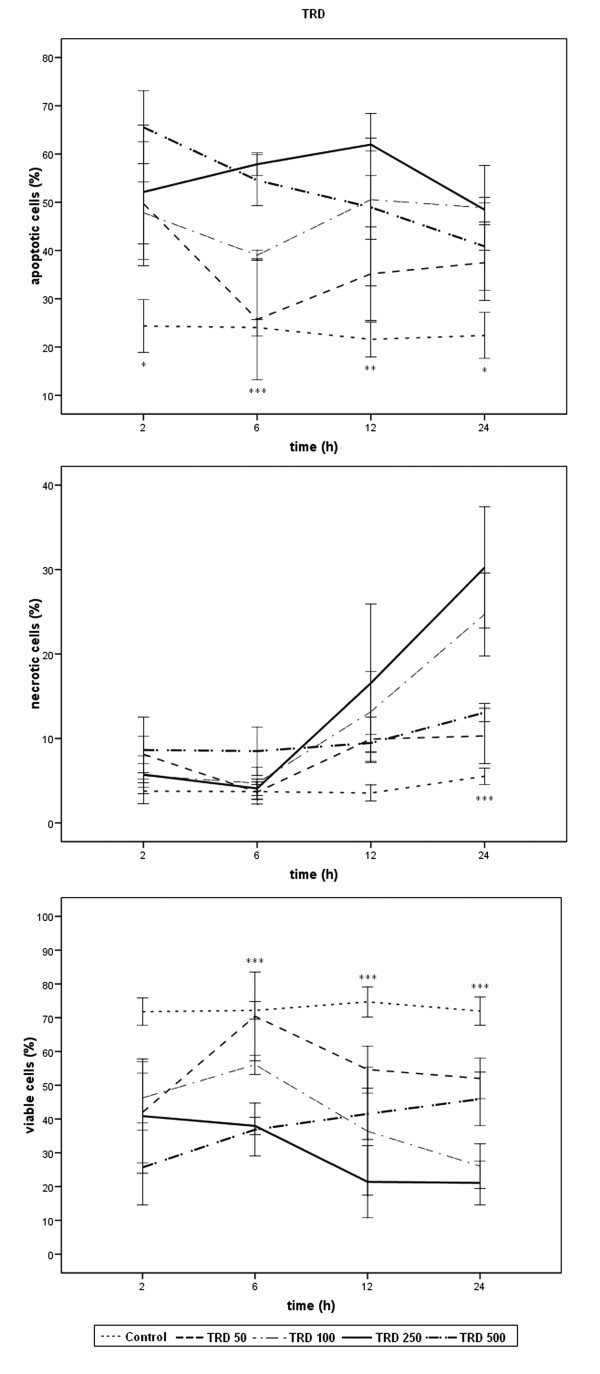
Effects of TRD on viability, apoptosis and necrosis in HT1080 cells measured by FACS-analysis: Cells were incubated with TRD in the concentrations indicated and with Povidon 5% (control) for 24 h. The percentages of viable, apoptotic and necrotic cells were determined by FACS-analysis for Annexin V-FITC and Propidiumiodide. Values are means ± SD of 3 independent experiments with consecutive passages. (*** p ≤ 0.001, ** p ≤ 0.005, * p < 0.05; one way ANOVA). The indicators of significance refer to the difference between the 250 μmol/l and the control series. The scales of the y-axis were adjusted to the different values for clarity and therefore vary.

**Figure 3:**
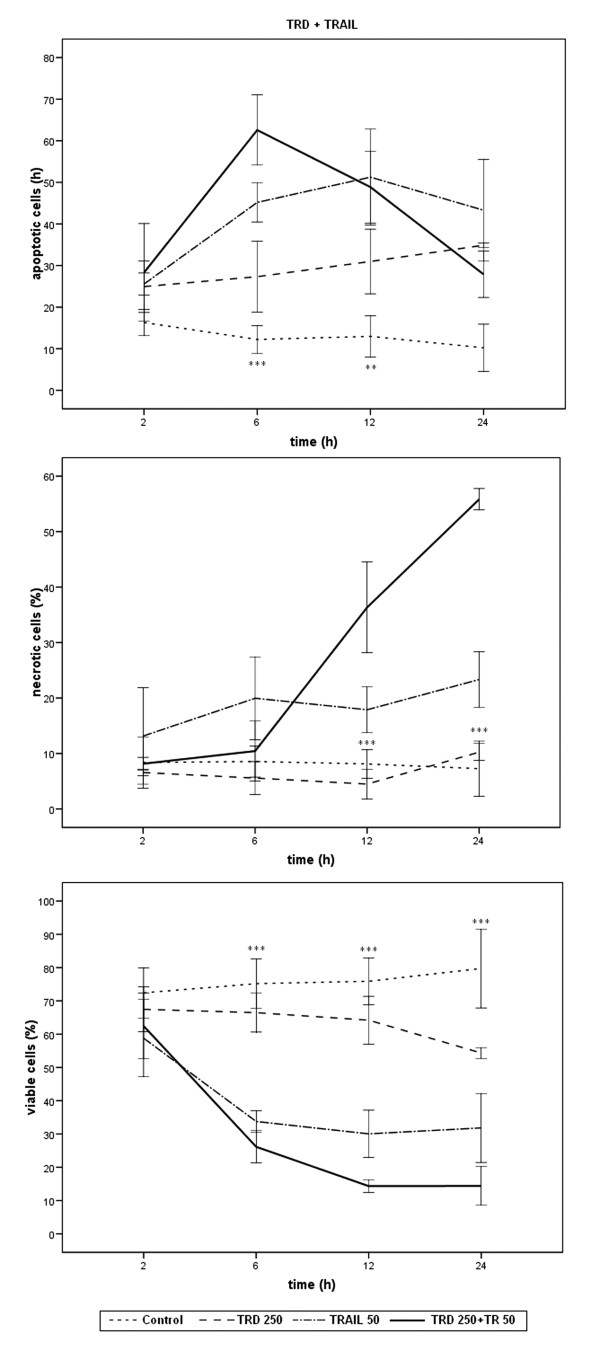
Effects of TRD, TRAIL and combination of both agents on viability, apoptosis and necrosis in HT1080 cells measured by FACS-analysis: Cells were incubated with 250 μmol/l TRD and 50 ng/ml TRAIL alone and in combination as well as with Povidon 5% + H_2_O (control) for 2 h, 6 h, 12 h, and 24 h. The percentages of viable, apoptotic and necrotic cells were determined by FACS-analysis for Annexin V-FITC and Propidiumiodide. Values are means ± SD of 3 independent experiments with consecutive passages (*** p ≤ 0.001, ** p ≤ 0.005; one way ANOVA). The indicators of significance refer to the difference between the TRD 250 μmol/l + TRAIL 50 ng/ml and the control series. The differences in the values compared to the single dose experiments are caused by experimental variability. The scales of the y-axis were adjusted to the different values for clarity and therefore vary.

Results of FACS-analysis for percentages of viable, apoptotic and necrotic cells are expressed as means ± SD of at least three independent experiments with consecutive passages. In this study, comparisons between experimental groups (single agent application in different doses and single agents versus combined treatment at various time points) were performed using one way measures of variance (one way ANOVA) over all time points (Tukey). P-values ≤ 0.05 were considered as statistically significant and indicated in the figures as follows: *** p ≤ 0.001, ** p ≤ 0.005, * p ≤ 0.05. The indications in figures 1, 2, 3 refer to TRD 250 μmol/l, TRAIL 50 ng/ml, and TRD250 μmol/l+TRAIL50 ng/ml compared to the control.

### Oligonucleotide microarray analysis

To identify the changes on gene expression level caused by the treatment with TRAIL and TRD, total RNA was purified from the cells after incubation with the different substances for 2 h using the RNeasy KIT from Qiagen (Hilden, Germany), as specified by the manufacturer. RNA integrity was assessed using the Agilent 2100 Bioanalyzer (Agilent Technologies).

For microarray analyses, we used the Affymetrix GeneChip platform employing a standard protocol for sample preparation and microarray hybridization. Total RNA (5 μg) was converted into biotinylated cRNA according to the Affymetrix standard protocol version 2, purified, fragmented and hybridized to HG-U133Plus_2.0 microarrays (Affymetrix). The arrays were washed and stained according to the manufacturer's recommendation and finally scanned in a GeneChip scanner 3000 (Affymetrix).

Array images were processed to determine signals and detection calls (Present, Absent, Marginal) for each probeset using the Affymetrix GCOS1.4 software (MAS 5.0 statistical algorithm). Arrays were scaled across all probesets to an average intensity of 1000 to compensate for variations in the amount and quality of the cRNA samples and other experimental variables of non-biological origin. Pairwise comparisons of treated *versus *control samples were carried out with GCOS1.4, which calculates the significance (change p-value) of each change in gene expression based on a Wilcoxon ranking test. To limit the number of false positives, we restricted further target identification to those probesets, which received at least one present detection call in the treated/control pair. Probesets exhibiting a significant increase or decrease were identified by filtering using the Affymetrix Data Mining Tool 3.0.

### Real-time PCR for microarray data validation

Microarray data validation was performed for selected candidate genes (*ARGHGDIA, BIRC3, GADD34, HSPA1A, HSPA1B, MAP3K14, MAP3K1*). These were identified as the most differentially regulated ones in microarray analysis. Total RNA (2 μg) was reverse transcribed using the High Capacity cDNA Archive Kit (Applied Biosystems). Realtime PCR was done with a 7900 HT SDS system (Applied Biosystems) in 20 μl reaction volume containing 1× Master Mix, 1 μl assay and cDNA equivalent to 2 ng total RNA. All reagents and realtime PCR assays (*ARGHGDIA *Hs00976924 g1, *BIRC3 *Hs00154109 m1, *GADD34 *Hs00169585 m1, *HSPA1A *Hs00359163 s1, *HSPA1B *01040501 +sH, *MAP3K14 *Hs01089753, *MAP3K1 *Hs00394890 m1) used were purchased from Applied Biosystems. Reactions were performed in duplicates and analysed by the deltadeltaCT method. Human *GAPD *was used for normalization.

### Western Blot

To validate the findings of changed gene expression on protein level, Western Blots were performed using an SDS-page gel and the following antibodies (rabbit): *Rho GDIα/ARGHGDIA *(C-21), *GADD 45α *(H-165), *c-IAP2*/*BIRC3 *(H-85), *GADD 34/PPP1R15A *(S-20), *NIK/MAP3K14 *(H-248), and *IκB-α/NFKBIA *(C-21), purchased from Santa Cruz Biotechnology Inc. (Heidelberg, Germany). Total protein was purified from the cells after incubation with the different substances for two different time points (2 h and 4 h); for this purpose floating cells were collected together with the supernatant, adherent cells were harvested by trypsinization and added to the solution. Cells were sedimented by centrifugation. After removal of the supernatant, the probes were incubated with 50 μl Cell Culture Lysis Reagent (Promega Corporation, Mannheim, Germany) each for 1 h on ice; the cell remnants then sedimented by centrifugation and the supernatant containing the purified protein deep frosted until further use.

Nuclear extract was derived from cells treated with the different substances for 4 h using the Nuclear Extract Kit (Aktive Motif Europe, Rixensart, Belgium) according to the manufacturer's instructions.

### *NFKB*-activity-ELISA

The transcription factor *NFKB *activity was detected and quantified using the TransAM™ *NFKB *Family Transcription Factor Assay Kit (Aktive Motif Europe, Rixensart, Belgium) according to the manufacturer's instructions and analyzed using a microplate absorbance reader Sunrise™ (Tecan trading AG, Switzerland). Nuclear extract, derived as specified above from cells treated with the different substances for 2 hours, was applied on a 96-well plate, to which oligonucleotide containing an *NFKB *consensus binding site has been immobilized. Activated *NFKB *specifically binds to these and was detected by using a primary antibody that is directed against the subunits *p50*, *p52*, *p65*, *c-Rel*, and *RelB*. An HRP-conjugated secondary antibody provided a colorimetric readout that was spectophotometrically analysed.

## Results

HT1080 fibrosarcoma cells are TRAIL sensitive. TRAIL as single agent caused apoptotic cell death time and dose dependently. TRAIL 100 and 500 ng/ml significantly induced early apoptosis after 2 h and resulted in a significant increase of necrotic cells at the following time points. TRAIL 50 ng/ml, with 77.1% apoptotic cells, after 6 h reached almost the same efficiency concerning apoptosis but did not lead to comparable necrosis rates. Additionally, the effect on apoptosis lasted longer than in the 100 and 500 ng/ml group. At no time point did the percentage of necrotic cells significantly exceed the rates of necrotic cells of the control group. The lowest TRAIL concentration used (10 ng/ml) was significantly less effective in reducing viable cells than the other three concentrations (fig. 1). Therefore, TRAIL 50 ng/ml was used for the combination therapy.

TRD also induces apoptotic cell death in human HT1080 fibrosarcoma cells. The apoptogenic and necrosis inducing effects were dose and time dependent. The highest rate of apoptotic cells (after 12 h, 62.0%) and the most effective reduction of viable cells (after 12 h, 21.1%) was seen at a single concentration of 250 μmol/l. The apoptotic and necrotic effects of TRD 50 μmol/l were only moderate and did not reach significance at any time point compared to the control. TRD 100 and TRD 500 μmol/l also induced significant apoptotic cell death but were less effective than the 250 μmol/l concentration over all time points. (fig. 2). Therefore, TRD 250 μmol/l was chosen to be applied together with TRAIL 50 ng/ml.

### Combination of TRAIL 50 ng/ml and TRD 250 μmol/l

After 6 h a significant increase of apoptotic cells (62.6%) was detected compared to TRAIL (p = 0.049) and TRD (p = 0.001) as single substances as well as compared to the control (p < 0.001), while necrosis was not significantly increased. After 12 h and 24 h, the necrotic proportion of cells increased and was significantly higher than in the single substances and the control group (p < 0.001), while the amount of apoptotic cells decreased again. The combination therapy reached the peak of apoptotic rates earlier and could transduce apoptotic cells to cell death more quickly, thereby reducing the proportion of viable cells more effectively (after 12 h, viable cells: 14.3%) than the single substances. TUNEL assay and cytochemistry also showed qualitatively against the control that cells treated with TRD/TRAIL, TRAIL, and TRD underwent apoptotic cell death (data not shown).

### TRD and TRD/TRAIL induce morphological changes and cell detachment

**Figure 4:**
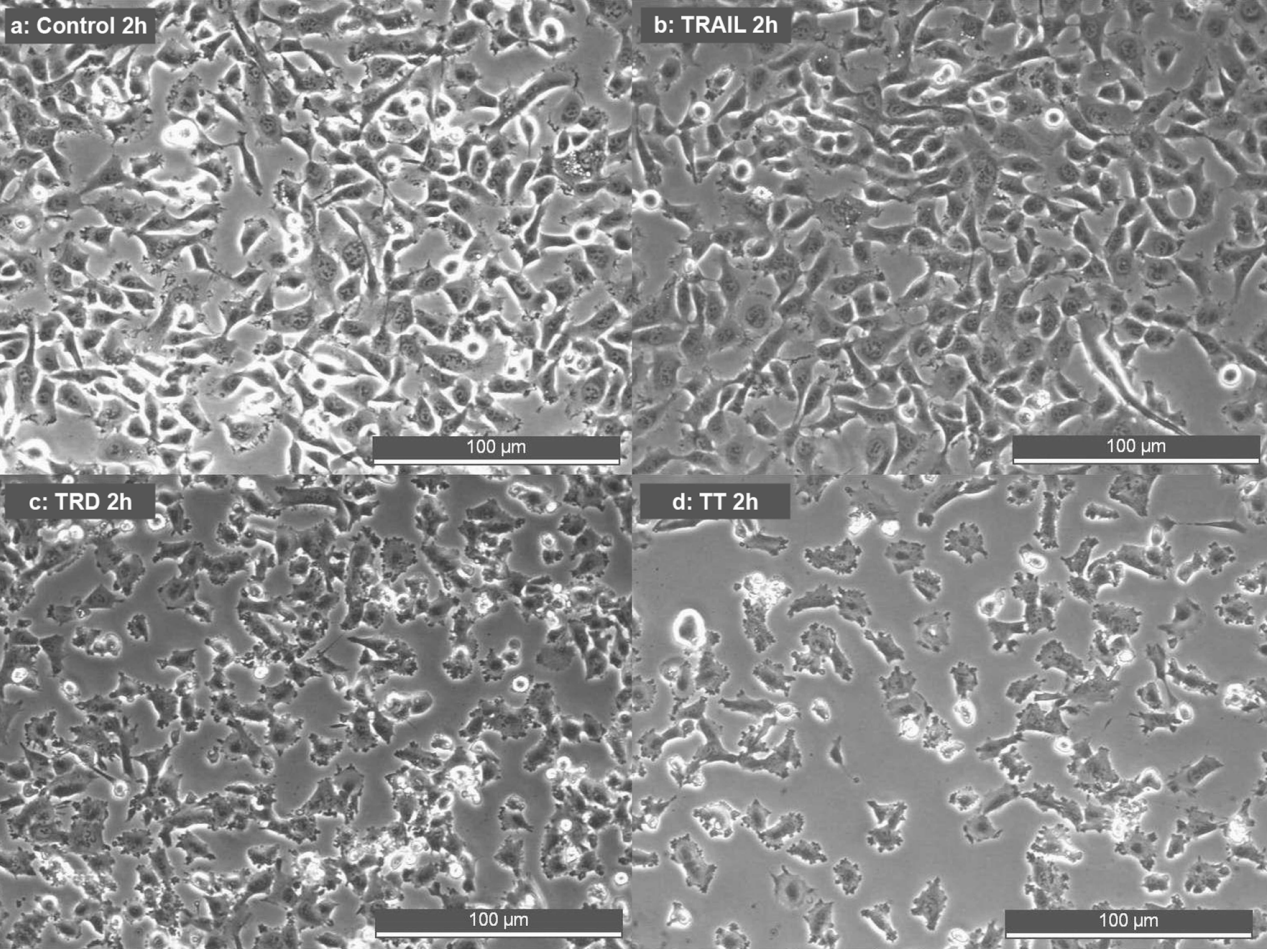
Phase contrast microscopic photographs showing morphologic changes induced by TRD, TRAIL and combination of both agents after 2 h: Cells were incubated with Povidon 5% + H_2_O (control) (a), 50 ng/ml TRAIL (b), 250 μmol/l TRD (c) and a combination of TRD/TRAIL (d).

TRAIL incubation did not change the cells' morphology and did not cause a detachment of the cells from the ground as shown by bright-field microscopy. TRD as single substance and the combination of TRD and TRAIL resulted in shrinkage of the cells, followed by complete cell detachment (fig. 4).

**Figure 5:**
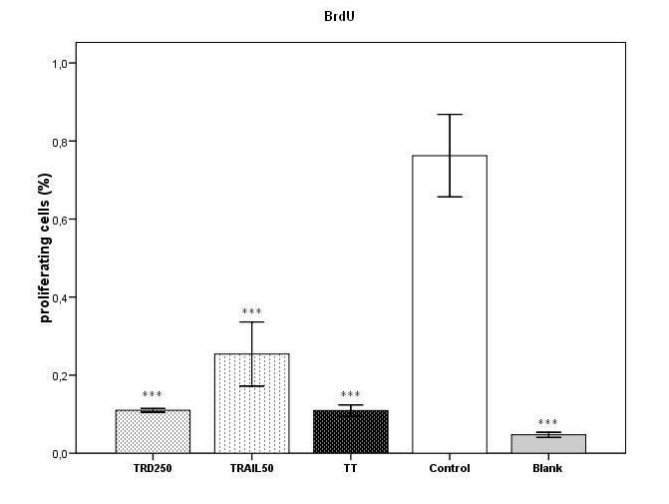
Effects of TRD, TRAIL and combination of both agents on proliferation were measured by BrdU cell proliferation-assay. Cells were incubated for 8 h with Povidon 5% + H_2_O (control) (a), 50 ng/ml TRAIL (b), 250 μmol/l TRD (c) and a combination of TRD/TRAIL (d). A blank negative control (e) was used to document absence of proliferation.

TRD, TRAIL and the combination therapy reduced proliferation of HT1080 significantly compared to the control (p < 0,001) as indicated by the BrdU-Assay (fig. 5). The combination therapy could not add to this effect compared to the incubation with TRD alone (p = 1.0), but reduced proliferation significantly compared to TRAIL alone (p < 0.001). The anti-proliferative potency of TRD was significantly higher than that of TRAIL (p < 0.001).

#### Gene expression

**Figure 6:**
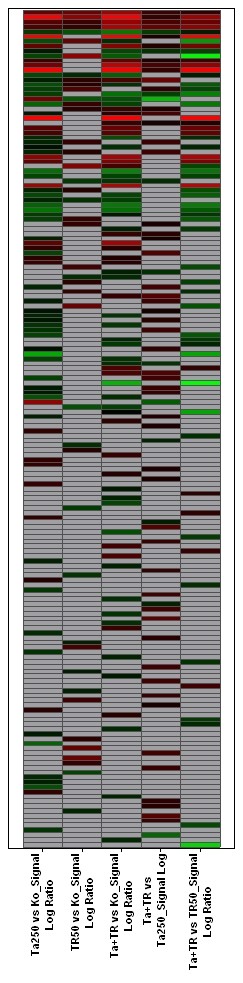
Overall expression patterns of 174 reliably measured probe sets associated with apoptosis out 621 apoptosis associated probe sets of the HG-U133A_2.0 chip. Horizontal rows represent individual probe sets/genes; vertical columns represent individual samples (from left to right: Colour range: Brightest red: Signal Log Ratio (SLR) >= 2 (indicates expression level above compared sample); brightest green: SLR <= 2 (indicates expression level below compared sample); black: SLR = 0 (indicates unchanged expression); grey: no reliable filter target.

**Figure 7:**
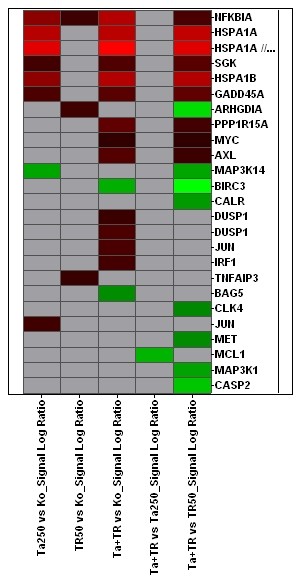
Overall expression patterns of 25 reliably measured probe sets associated with apoptosis out 621 apoptosis associated probe sets of the HG-U133A_2.0 chip. Horizontal rows represent individual probe sets/genes; vertical columns represent individual samples (from left to right: Colour range: Brightest red: Signal Log Ratio (SLR) >= 2 (indicates expression level above compared sample); brightest green: SLR <= 2 (indicates expression level below compared sample); black: SLR = 0 (indicates unchanged expression); grey: no reliable filter target.

In this experiment we selectively focussed on apoptosis related probesets. Out of 621 of those probesets, 174, representing 138 apoptosis related genes, showed expression changes (fig. 6). TRD alone induced differences in the expression of 67 apoptosis related genes, of which 22 were "upregulated"; by comparison, TRAIL as a single substance caused expression changes of 36 genes related to apoptotic pathways, "upregulating" 22 of them. TRD and TRAIL in combination induced changes in the expression of 65 genes (29 upregulated, 28 downregulated) A list of these genes, including the log ratios of the expression changes, is given in table 1. Further filtering of the results, leaving only the genes whose expression changes that had a signal log ratio above 1 or below -1, reduced the number of differentially regulated genes in this experiment to 21 (25 probe sets) (fig. 7). The expression of selected candidate genes was re-evaluated by rtPCR, revealing a largely good correlation (table 2a/b).

**Table 1:** Additional information about the genes whose expression was changed more than two-fold in the experiments.

Gene Symbol	Ta250 vs Co Signal Log Ratio	Gene Symbol	TR50 vs Co Signal Log Ratio	Gene Symbol	Ta+TR vs Co Signal Log Ratio	Gene Symbol	Ta+TR vs Ta250 Signal Log Ratio	Gene Symbol	Ta+TR vs TR50 Signal Log Ratio
HSPA1A/B	2,99	ARHGDIA	1,19	HSPA1A/B	3,28	TIE1	0,89	HSPA1A/B	2,96
NFKBIA	2,03	NFKBIA	1,17	NFKBIA	2,47	AXL	0,85	GADD45A	1,55
GADD45A	1,33	TNFAIP3	1,11	PPP1R15A	1,55	IRF2	0,76	SGK	1,45
SGK	1,22	JUN	0,89	GADD45A	1,46	ERBB2	0,71	NFKBIA	1,3
JUN	1,2	EGFR	0,86	AXL	1,41	RELA	0,69	PPP1R15A	1,22
PPP1R15A	0,95	CALR	0,85	SGK	1,37	TIAF1	0,69	AXL	1,16
MCL1	0,94	TP53	0,64	DUSP1	1,33	TIMP3	0,67	MYC	1,01
DUSP1	0,82	TNK2	0,58	JUN	1,31	DUSP5	0,66	DUSP1	0,98
MYC	0,78	PPP2CB	0,57	IRF1	1,23	PPP1R15A	0,61	IRF1	0,84
BTG1	0,7	BAX	0,51	MYC	1,05	LITAF	0,6	BTG1	0,84
IRF1	0,69	IRF1	0,47	BTG1	0,85	IRF1	0,6	BCL2A1	0,52
AXL	0,64	IKBKG	0,44	DUSP5	0,65	TP53	0,57	BAG1	0,46
RPS3A	0,48	BCL2L1	0,44	TIE1	0,63	TYRO3	0,55	NEU1	0,41
LDHB	0,44	AXL	0,42	NEU1	0,63	LTBR	0,55	JUN	0,39
ESD	0,43	PPP2R1A	0,41	HD	0,63	TYK2	0,54	ANXA4	0,37
HSPD1	0,42	CD44	0,38	TNFAIP3	0,55	SIPA1	0,54	GSTP1	0,31
RPS3A	0,41	TIE1	0,37	CDKN1A	0,55	TRAF4	0,53	CROP	-0,24
NME1	0,41	DUSP3	0,37	ACTN4	0,5	DOCK1	0,53	CASP8	-0,24
LGALS1	0,4	ACTN4	0,34	EPHA2	0,48	CSK	0,52	PPM1D	-0,31
ENO1	0,38	PEA15	0,32	GSTP1	0,47	ACIN1	0,52	API5	-0,32
STK17A	0,3	DAPK3	0,31	PPM1G	0,45	ARHGDIA	0,49	TNFRSF10B	-0,34
API5	-0,14	ACIN1	0,31	TIAF1	0,44	ABCA2	0,48	FOXO1	-0,37
DDR2	-0,15	BID	-0,23	CFL1	0,4	TNFRSF25	0,47	RAD21	-0,38
PDCD4	-0,17	MAP3K5	-0,27	LITAF	0,37	TNFRSF1A	0,47	EGFR	-0,39
E2F1	-0,18	FER	-0,27	PINK1	0,36	FGFR1	0,47	DUSP10	-0,45
LITAF	-0,2	FAS	-0,3	RPS3A	0,35	DNM2	0,47	NFKB1	-0,46
EPHB2	-0,21	CROP	-0,33	LGALS1	0,32	NFKBIA	0,45	FOXO3	-0,46
FGFR1	-0,22	HSPA9	-0,37	PPP1CA	0,29	EPHB2	0,45	RYBP	-0,49
RIPK1	-0,22	CHUK	-0,4	ENO1	0,28	IRF3	0,42	MCL1	-0,49
CSK	-0,25	LYN	-0,43	MAP3K1	-0,04	DDR2	0,42	DUSP3	-0,53
TIE1	-0,25	CUL2	-0,47	DUSP10	-0,21	WEE1	0,38	BCLAF1	-0,56
TNFRSF21	-0,25	TIA1	-0,49	FAS	-0,22	E2F1	0,36	TP53	-0,58
ACIN1	-0,28	PPM1B	-0,55	MCL1	-0,26	BAK1	0,36	HELLS	-0,61
PPP3CB	-0,28	CASP8	-0,68	CUL4A	-0,26	PPM1G	0,34	PPP2CB	-0,62
CHUK	-0,29	MCL1	-0,7	FADD	-0,27	FYN	0,31	DAPK3	-0,64
DAPK3	-0,29			TIA1	-0,28	RYK	0,3	TNFAIP3	-0,67
PPP2R1B	-0,29			PAWR	-0,29	DUSP1	0,3	SOCS2	-0,71
YWHAH	-0,29			PPP3CC	-0,31	ACTN4	0,3	CLK1	-0,72
RYK	-0,34			FOXO3	-0,32	CTSB	0,28	IKBKG	-0,74
MAP3K11	-0,35			TIA1	-0,34	HSPA1A/B	0,25	BAG5	-0,97
BCLAF1	-0,36			RIPK2	-0,34	SART1	0,24	BCL2L1	-0,99
DOCK1	-0,4			SOCS2	-0,37	DHCR24	0,24	CLK4	-1,02
F2R	-0,4			CASP7	-0,37	JUN	0,23	MET	-1,05
SIAH1	-0,41			CALR	-0,37	PPP4C	0,22	CALR	-1,23
AHR	-0,44			WEE1	-0,38	PPP2R1A	0,21	MAP3K1	-1,32
CASP8	-0,47			API5	-0,39	GSTP1	0,21	MAP3K14	-1,34
DUSP10	-0,48			MAP2K4	-0,41	YWHAH	0,2	CASP2	-1,7
MET	-0,51			ABL2	-0,42	PDCD4	0,18	ARHGDIA	-1,95
TP53	-0,52			CUL2	-0,44	FXR1	-0,24	BIRC3	-2,32
ABL2	-0,54			PPP2R1B	-0,45	PHB	-0,28		
IKBKG	-0,54			SIAH1	-0,46	YES1	-0,29		
RELA	-0,54			PPM1D	-0,46	CUL2	-0,38		
ARHGDIA	-0,55			CHUK	-0,5	CUL4A	-0,39		
SOCS2	-0,55			BCL2L1	-0,54	CASP7	-0,42		
TIMP3	-0,55			TIA1	-0,56	BCLAF1	-0,42		
CASP2	-0,59			CASP2	-0,56	CASP8	-0,49		
CROP	-0,59			RYBP	-0,63	MET	-0,53		
PRF1	-0,64			BCLAF1	-0,64	TFG	-0,84		
CALR	-0,65			ARHGDIA	-0,74	MCL1	-1,5		
WEE1	-0,66			CROP	-0,78				
ERBB2	-0,8			CLK1	-0,88				
CLK4	-0,81			MET	-0,91				
BCL2L1	-0,82			CASP8	-0,97				
BAG5	-0,9			CLK4	-0,99				
CLK1	-0,92			BAG5	-1,08				
MAP3K14	-1,35			BIRC3	-1,45				

**Table 2:** 

**a: Summary of the selected candidate genes' microarray data (/stands for no value).**											
Microarray Probeset	201167_x_at	201168_x_at	210538_s_at	202014_at	203725_at	200799_at	200800_s_at	202581_at	214786_at	205192_at	201502_s_at

Gene Symbol SLR = log2(RQ)	ARHGDIA	ARHGDIA///LOC728908	BIRC3	PPP1R15A/Gadd34	GADD45A	HSPA1A	HSPA1A///HSPA1B	HSPA1B	MAP3K1	MAP3K14	NFKBIA

Ta+TR vs Ko_SLR	-0,74	/	-1,45	1,55	1,46	2,54	3,28	2,45	-0,04	/	2,47

TR50 vs Ko_SLR	1,19	0,43	/	/	/	/	/	/	/	/	1,17

Ta250 vs Ko_SLR	-0,55	-0,28	/	0,95	1,33	2,49	2,99	2,06	/	-1,35	2,03

Ta+TR vs TR50_SLR	-1,95		-2,32	1,22	1,55	2,61	2,96	2,44	-1,32	-1,34	1,3

Ta+TR vs Ta250_SLR	/	0,49	/	0,61	/	/	0,25	0,43	/	/	0,45

**b: Summary of the selected candidate genes' rtPCR data. The correlation with the findings of the microarray is high except for ARGHGDIA.**											

Taqman assay	Hs00976924_g1	Hs00154109_m1	Hs00169585_m1	Hs00169255_m1	Hs00359163_s1	Hs01040501_+sH	Hs01089753	Hs00394890_m1	Hs00153283_m1		

Gene Symbol SLR = log2(RQ)	ARGHGDIA	BIRC3	GADD34	GADD45A	HSPA1A	HSPA1B	MAP3K14	MAP3K1	NFKBIA		

TA+Tr vs Control	-0,4205	-1,1638	1,2819	2,4504	3,8893	3,1561	-0,8706	-0,5410	2,3151		

TR50 vs Control	-0,1207	1,1527	0,2107	0,7523	0,4395	0,4545	0,1833	0,0203	1,5287		

TA25 vs Control	0,0120	-0,3926	1,4327	2,8888	3,6653	3,5939	-0,7547	-0,1194	2,5517		

TA+Tr vs TR50	-0,2998	-2,3165	1,0712	1,6981	3,4498	2,7016	-1,0539	-0,5613	0,7864		

TA+Tr vs TA25	-0,4325	-0,7712	-0,1508	-0,4385	0,2240	-0,4378	-0,1159	-0,4217	-0,2366		

**Figure 8:**
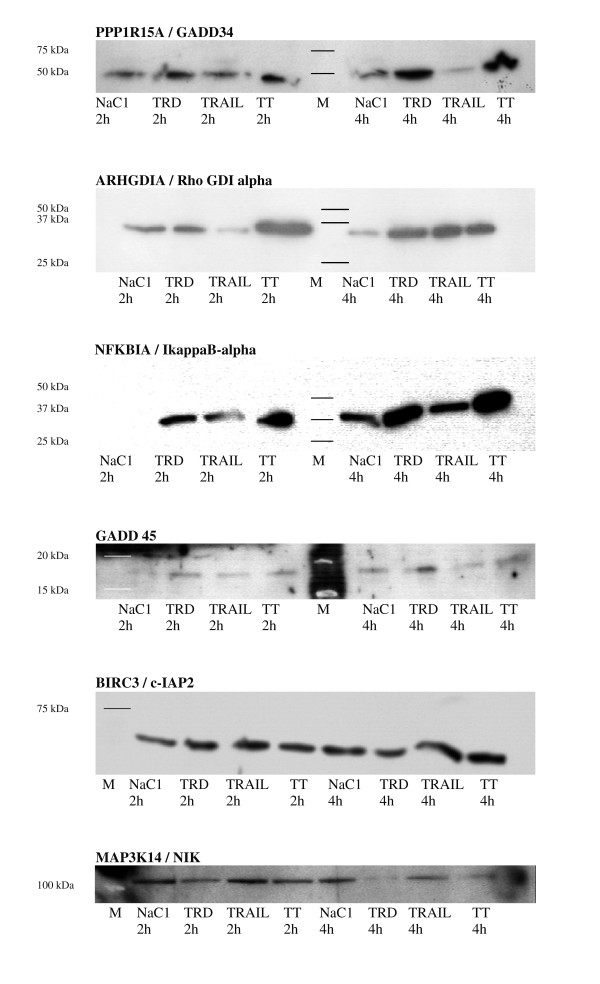
Representative Western Blot results for selected cytosolic proteins (M = marker, indicated by lines) after 2 and 4 h.

Further analysis of the translation of the candidate genes after 2 and 4 h tested in the rtPCR was performed by Western Blot. The results are presented in figure 8. The results for *NFKBIA*, *PPP1R15A, MAP3K14*corresponded well to the rtPCR and microarray data, whereas the findings for *ARHGDIA *were partly oppositional to them. The other proteins (*GADD45A*, *BIRC3*) showed no such noticeable changes.

**Figure 9:**
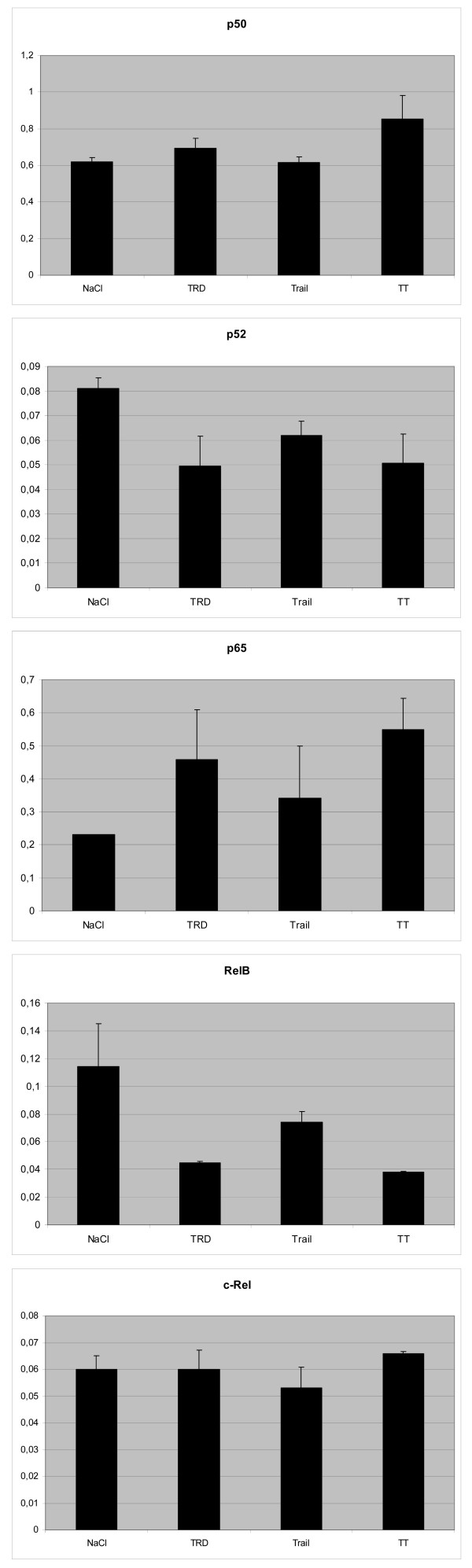
Diagram showing the results of the *NFKB*-ELISA including the standard deviation of three separate measurements.

The transcription factor *NFKB *activity was measured by quantifying its subunits *p50*, *p52*, *p65*, *c-Rel*, and *RelB*. The results for the several subunits are illustrated in figure 9. Although the changes of the single substances and the combination therapy compared to the control were not significant, it is noteworthy that the changes of TRD and TRAIL/TRD were always more pronounced than those of TRAIL as single substance.

## Discussion

To date, several reports suggested TRAIL as a promising substance in the treatment of sarcoma, especially when combined with other cytostatics.

Several studies found, that TRAIL was effective inducing apoptotic cell death and that the combination therapy of TRAIL and doxorubicin could overcome TRAIL resistance in a variety of soft tissue sarcoma cells [22].

In this study, TRAIL as a single substance effectively induced apoptotic cell death in HT1080 fibrosarcoma cells. Notably, only three genes (*ARHGDIA*, *TNFAIP3*, *NFKBIA*) were differentially up-regulated more than two-fold compared to the control: *ARHGDIA *(Rho Guanosine Diphosphate-Dissociation Inhibitor A), that inhibits dissociation of Guanosine Diphosphate (GDP) from *RhoA*, thereby preventing it from binding GTP (Guanosine Triphosphate) and inactivating it. *RhoA*, an important regulator of the cytoskeleton, cell adherence and cell motility, is associated to the occurrence of metastases in several tumors [23,24]. In HT1080 cells that show high levels of Rho-GTP the inhibition of *Rho *by fasudil, a Rho kinase inhibitor, leads to decreased tumor cell motility and growth [25]. The findings that gene expression of *ARHGDIA *was decreased by the combination therapy and by TRD, whereas the protein could be detected at much higher cytosolic concentrations after treatment with TRAIL and TRD, so far cannot be explained but may be the reason for cell detachment and the changes in cell morphology. The fact that the Western Blot findings after 4 h corresponded much better to the findings after 2 h may be caused by the time translation takes to increase cytosolic protein concentration.

*TNFAIP3*, that was found to be up-regulated by TRAIL in our study, is an inhibitor of the *NFKB *pathway [26] and may thereby promote apoptosis but, on the other hand, it was shown to decrease *TNF*-mediated apoptosis and necrosis [27], leaving its specific influence unclear.

*NFKBIA *is an inhibitor of *NFKB*, which has been associated with resistance to chemotherapeutics such as doxorubicin [28]. The doxorubicin analogon DA-125 could reduce proliferation in HT1080 fibrosarcoma cells through a *NFKB *dependent pathway [29] and recent studies showed that tumor invasiveness could be significantly reduced by inhibiting *NFKB *activity [30], pointing to this transcription factor as a key element in HT1080 proliferation-pathways. In our study, NFKBIA was found to be over-expressed on gene and on protein level after TRAIL, TRD and the combination therapy.

As previously reported, TRD lacks toxic short or long term effects but has the ability to induce apoptotic cell death in a variety of malignant cells. In several osteosarcoma cell lines, TRD has already been shown to induce apoptosis and decrease cell adhesion [31], but to the authors' knowledge TRD or the combination of TRAIL and TRD has not yet been investigated on soft tissue sarcoma. Interestingly, Taurolidine was shown to reduce toxicity of *TNF *in vivo without reducing its antitumoral activity; probably by interfering not with *TNF *directly but with its downstream pathway, which is largely the same for TRAIL [16,32], qualifying this substance for co-treatment with TRAIL.

The changes in cell morphology and detachment of the cells from the ground that were observed after incubation with TRD may be explained by the finding of other studies; TRD reduced the expression of integrins and cadherins in colon cancer cells and reduced intraperitoneal metastases and tumor growth accordingly [33].

Gene expression profiling revealed a small number of genes whose expression was changed more than two-fold. Among them, the heat shock protein *HSPA1A/B*. Increased expression of this protein was associated with increased chemosensitivity of HT1080 to mitomycin C [34]. Furthermore, the apoptogenic effects of taxanes on sarcoma could be increased by co-therapy with STA-4783, a stimulator of *HSPA1A *expression [35]. *NFKBIA*, that was also up-regulated by TRD, has already been mentioned. Notably, there are reports that TRD inhibits the activation of *NFKB *not only indirectly through *NFKBIA *but, also by direct interference, by oxidation of *NFKB *at Met45 [36].

Upregulation of *GADD45A *was shown to be associated with increased apoptosis and *p53 *independent cell cycle arrest in a variety of soft tissue sarcomas [37].

It inhibits transcription factors associated with tumor growth such as *JNK *(*c-Jun N-terminal kinase*) and *NFKB *[38,39]. For rhabdomyosarcoma, increased *GADD45A *was associated with less aggressive tumor behaviour [40]. Additionally *GADD45A *may antagonize TNF-receptor mediated cytotoxicity [41]. *SGK*, that was also found to be up-regulated in contrast to most of the other differentially expressed genes can activate the *NFKB *pathway and thereby prevent cells from undergoing apoptosis [42]. In this context, this effect seems to be outweighed by other proapoptotic ones. *JUN *is activated *JNK *dependently and promotes apoptotic cell death in malignant cells including osteosarcoma [43]. Downregulation of *JUN *was shown to decrease the expression of matrix metalloproteinases and thereby cellular invasiveness in HT1080 cells [44]. This downregulation may be mediated through suppression of *NFKB *activation [45]. *MAP3K14*, the only gene that was down-regulated more than two-fold by TRD, is a member of the TNF-pathway and activates *NFKB *(IKKalpha) [46].

The significant increase in apoptosis and necrosis using the combination of the two substances was accompanied by a large number of expression changes. Therefore, we will not further discuss the ones that were already described in the TRAIL and TRD section of the discussion; we summarized the remaining genes and their functions in table 3. Interestingly, there was only one gene with two-fold expression changes when the TRD/TRAIL cells were compared to those that were incubated with TRD alone: *MCL1*, that is expressed in a variety of soft tissue sarcomas and acts anti-apoptotic, was down-regulated [47].

**Table 3:** Summary of the expression changes of apoptosis related genes for the single substances (TRD 250 μmol/l, TRAIL 50 ng/ml) compared to untreated cells and the combination therapy compared to Control, TRD and TRAIL treated cells.

Gene Symbol	Gene Title	Synonyms	Gene function
NFKBIA	nuclear factor of kappa light polypeptide gene enhancer in B-cells inhibitor, alpha	IkappaB-alpha, IkB-a, IkBa, Inhibitor of kappa B-alpha, MAD3, P40	Proliferation in HT1080 cells is mediated through a NFKB dependent pathways [29,54]. Tumor invasiveness could be significantly reduced in HT1080 cells by reducing NFKB activity [30]. Increased NFKB activity leads to doxorubicin resistance in a p53 dependent manner [28].
HSPA1A/B	heat shock 70 kDa protein 1A/B	HSP70, HSP72, HSPA1	Upregulation of HSPA1A significantly increased chemosensitivity of HT1080 to mitomycin C [34]. The apoptogenic effects of taxanes on sarcoma could be increased by co-therapy with stimulators of HSPA1A expression [35].
SGK	serum/glucocorticoid regulated kinase	serine/threonine-protein kinase Sgk1, serum/glucocorticoid-regulated kinase 1, SGK1	SGK activates the NFKB pathway and thereby can prevent cells from undergoing apoptosis [42].
GADD45A	growth arrest and DNA-damage-inducible, alpha	DDIT1, DNA-damage-inducible transcript 1, GADD45, Growth arrest and DNA-damage-inducible protein, GADD45 alpha	Upregulation of GADD45A is associated with increased apoptosis and cell cycle arrest p53 independently in a variety of soft tissue sarcomas [37]. It inhibits transcription factors associated with tumor growth including the c-Jun N-terminal kinase (JNK) cascade and *NFKB *[38,39,41,55]. For rhabdomyosarcoma, increased *GADD45A *expression was associated with less aggressive tumor behaviour [40]. GADD45 may antagonize TNF-receptor mediated cytotoxicity [41].
ARHGDIA	Rho GDP dissociation inhibitor (GDI) alpha	GDIA1, MGC117248, RHOGDI, Rho GDI 1, Rho-GDI alpha, Rho GDP-dissociation inhibitor 1	High levels of Rho-GTP are detected in HT1080 cells. The inhibition of Rho by fasudil, a Rho kinase inhibitor lead to decreased tumor cell motility and growth in HT1080 cells [25] and associated to the development of metastases in several other malignant tumors [23,24]. ARHGDIA is downregulated by doxorubicin in HT1080 cells [56].
PPP1R15A	protein phosphatase 1, regulatory (inhibitor) subunit 15A	GADD34, MyD116	Increased expression of PPP1R15A by chemosensitizers can potentiate the effects of cytostatics such as platinum agents [57] and probably acts p53 independently [58].
MYC	v-myc myelocytomatosis viral oncogene homolog (avian)	c-Myc, Myc proto-oncogene protein, transcription factor p64	Myc induces apoptosis by increasing the p53 levels JNK-dependently [59].
AXL	AXL receptor tyrosine kinase	oncogene tyrosine-protein kinase receptor UFO precursor, UFO	AXL is associated with metastatic potential of malignant cells by regulating adherence, motility, and invasiveness [60]. It can prevent cells from TNFalpha mediated cell death via the phosphatidylinositol 3-kinase [61] and the NFKB pathway [62].
MAP3K14	mitogen-activated protein kinase kinase kinase 14	FTDCR1B, HS, HsNIK, HSNIK, mitogen-activated protein kinase kinase kinase 14, NF-kappa beta-inducing kinase, NIK, serine/threonine-protein kinase NIK	MAP3K14 is a member of the TNF-Pathway and activates NFKB (IKKalpha) [46]. The MAPkinase pathway can induce apoptosis by induction of the GADD family of genes (GADD 34, GADD 45) [63].
BIRC3	baculoviral IAP repeat-containing 3	AIP1, API2, apoptosis inhibitor 2, Baculoviral IAP repeat-containing protein 3, cIAP2, CIAP2, C-IAP2, HAIP1, HIAP1, hiap-1, HIAP-1, IAP1, IAP homolog C, inhibitor of apoptosis protein 1, MALT2, MIHC, RNF49, TNFR2-TRAF signalling complex protein 1	BIRC3 is associated with chemotherapy resistance in Ewing sarcoma, rhabdomyosarcoma [64] and prostatic cancer [65] and suppresses TNFalpha mediated cell death by preventing formation of TNF Receptor 1. It regulates pro-survival NFKB-signalling by promoting degradation of MAP3K14 [66].
CALR	calreticulin	calregulin, calreticulin precursor, cC1qR, CRP55, CRTC, ERp60, FLJ26680, grp60, HACBP, RO, SSA	Calreticulin belongs to the family of heat shock proteins and strongly binds to TRAIL [67]. Calreticulin is translocated to tumor cells' membranes after anthracyline therapy and stimulates the anti-tumor immune response [68].
DUSP1	dual specificity phosphatase 1	CL100, dual specificity protein phosphatase 1, dual specificity protein phosphatase hVH1, HVH1, MAP kinase phosphatase 1, MKP1, MKP-1, protein-tyrosine phosphatase CL100, PTPN10, VH1	DUSP inactivates MAP kinases [69] and can protect cells from apoptotic stimuli by chemotherapeutics [70].
JUN	v-jun sarcoma virus 17 oncogene homolog	activator protein 1, AP1, p39, proto-oncogene c-jun, transcription factor AP-1, V-jun avian sarcoma virus 17 oncogene homolog	Jun is activated by TRAIL JNK dependently and promotes apoptotic cell death in malignant cells including osteosarcoma [43]. Downregulation of JUN decreases the expression of matrix metalloproteinases and thereby cellular invasiveness in HT1080 cells [44]. This down-regulation may be mediated through suppression off NFKB activation [45]. JUN is known to be a product of MAP2K4-activation and to mediate apoptosis by several chemotherapeutics [55]. upregulation of HSPA1A and JUN expression Chemosensitivity of HT1080 to mitomycin C could significantly be increased by [34].
IRF1	interferon regulatory factor 1	MAR	IRF1 inhibits cell growth and induces apoptosis via activation of caspases 1 and 7 [71]. It inhibits NFKB-dependent activation of matrix metalloproteinase-9 (MMP9) [72].
TNFAIP3	tumor necrosis factor, alpha-induced protein 3	A20, MGC104522, MGC138687, MGC138688, Putative DNA-binding protein A20, TNFA1P2, Zinc finger protein A20	TNFAIP3 down-regulates the TNF-α-induced NFKB signalling pathway [26] and reduces TNF mediated apoptosis and necrosis [27].
BAG5	BCL2-associated athanogene 5	BAG-5, BAG family molecular chaperone regulator 5, KIAA0873	BAG family members inhibit Hsp70 and promote cell growth and survival [73].
CLK4	CDC-like kinase 4	Dual specificity protein kinase CLK4	CLK family members prevent cells from undergoing intrinsic apoptosis [74].
MET	met proto-oncogene (hepatocyte growth factor receptor)	c-Met, Hepatocyte growth factor receptor precursor, HGF/SF receptor, HGFR, HGF receptor, Met proto-oncogene tyrosine kinase, RCCP2, Scatter factor receptor, SF receptor	Over-expression of MET was associated with enhanced proliferation and aggressive tumor biology in sarcomas[75]. Survival, anchorage dependent growth and invasiveness of sarcoma cells are dependent on MET [76].
MCL1	Myeloid cell leukemia sequence 1 (BCL2-related)	Bcl-2-related protein EAT/mcl1, EAT, Induced myeloid leukemia cell differentiation protein Mcl-1, mcl1/EAT, MCL1L, MCL1S, MGC104264, MGC1839, TM	MCL1 is expressed in a variety of soft tissue sarcomas and acts anti-apoptotic. Inhibition of MCL1 in combination with low dose cyclophosphamide significantly increases apoptosis in HT1080 cells [47].
MAP3K1	mitogen-activated protein kinase kinase kinase 1	MAPK/ERK kinase kinase 1, MAPKKK1, MEKK, MEKK1, MEKK 1, MEK kinase 1	MEKK is activating MAPK and JNK. Reduction of MEKK activity amplifies the apoptotic effect of TNFalpha on fibrosarcoma cells [77].
CASP2	caspase 2, apoptosis-related cysteine peptidase (neural precursor cell expressed, developmentally down-regulated 2)	apoptosis-related cysteine peptidase (neural precursor cell expressed, developmentally down-regulated 2), CASP-2, Caspase-2 precursor, ICH1, ICH-1L, ICH-1L/1S, ICH-1 protease, NEDD2	Casp2 is a member of the caspases family and mediates apoptotic cell death NFKB and Jun dependently but independent from Fas [78].

Signal log ratios of the changes are given for the several samples (TRD vs control, TRAIL vs control, TRD/TRAIL vs control, TRD/TRAIL vs TRD), signal log ratio of 1 representing a twofold increase, one of -1, that the expression is half of the expression of the control group and so forth.

Careful interpretation of the data revealed that many of the genes involved point to the *NFKB *pathway. In physiological conditions, *NFKB *is sequestered in inactive form by inhibitory proteins like *NFKBIA *[48], that was found to be up-regulated by the tested substances in the microarray, the PCR and the Western Blot analysis. Activation of the *TNF-alpha *pathway was shown to be more efficient inducing apoptosis when the *NFKB *pathway was blocked simultaneously [49] and could be a reason for enhanced apoptosis in the co-treatment with TRAIL and TRD.

In recent studies, tumor invasiveness could be significantly reduced in HT1080 cells by reducing *NFKB *activity [30] and *NFKB *inhibition could sensitise cells to TNF mediated cell death, probably by inhibiting the inactivating effect on JNK [50].

*NFKB *is activated *TRADD*-, *TRAF3*- and *FADD*-dependently [38] and also plays a key role in the survival of tumor cells by inducing expression of anti-apoptotic genes such as *Bcl2*, *Bcl2l1*, vascular endothelial growth factor (*VEGF*), and X-linked inhibitor of apoptosis (*XIAP*). Out of TRADD, TRAF3, FADD, Bcl2, and Bcl2l1, microarray analysis in this study could only detect changed expression for *FADD and Bcl2l1*. FADD was down-regulated by the TRAIL and TRD combination and *Bcl2l1 *was up-regulated by TRAIL but down-regulated by TRD and the combination (tab. 1). The effect on *Bcl2l1 *may partly explain the synergistic effects of the substances' combination. The fact that *NFKB *induces the expression of cell adhesion molecules [51] could be a reason for disruption of cell adherence in our experiments.

In summary, taking the changes in gene expression, protein concentration and the results of the *NFKB *activity assay into account, the effects of TRD, TRAIL and the combination of the two substances seem to be closely related to *NFKB *and its associated pathways [8]. The changes in DNA binding activity of *RelA *(*p65*) and *RelB*, that was detected by the ELISA, may further support this assumption. *RelA*, *c-Rel *and *RelB *are known to contain C-terminal transcriptional activation domains (TADs), which enable them to activate target gene expression. In contrast, *p50 *that was increased and *p52 *that was found to be decreased, do not contain C-terminal TADs; therefore, *p50 *and *p52 *homodimers probably repress transcription unless they are bound to a protein containing a TAD, such as *RelA*, *c-Rel *or *RelB *[52,53]. Detailed knowledge about the function of these factors is scant though, especially concerning soft tissue sarcomas, so that we abstain from further interpretation.

## Conclusion

TRD and TRAIL are effective to induce apoptosis and decrease proliferation in human fibrosarcoma. A variety of genes seems to be involved, pointing to the NFKB pathway as key regulator in TRD/TRAIL-mediated apoptosis.

## Competing interests

The authors declare that they have no competing interests.

## Authors' contributions

AD: developed the study design, coordinated the work, interpreted the data and prepared the manuscript. CB: carried out the experiments and interpreted the data. DB: coordinated the FACS analysis and interpreted the data. AG: established cell culture, interpreted the literature and prepared the manuscript. CG: coordinated and conceived the microscopy and interpreted the data. ML: developed the study design and corrected the manuscript. HUS: was helpful in preparing the manuscript and conceived the work. AF: did the Western Blot analysis and coordinated the laboratory work. LS: prepared the figures and coordinated cell culturing. LKH: carried out and interpreted the microarrays. UM: developed the idea, improved the study design and coordinated the work. WU: gave substantial contribution to the manuscript and the study design. AMC: carried out statistical analyses, have given substantial contribution to conception and design as well manuscript preparation.
